# Applications of Surface Plasmon Resonance (SPR) for the Characterization of Nanoparticles Developed for Biomedical Purposes

**DOI:** 10.3390/s121216420

**Published:** 2012-11-27

**Authors:** Mara Canovi, Jacopo Lucchetti, Matteo Stravalaci, Francesca Re, Davide Moscatelli, Paolo Bigini, Mario Salmona, Marco Gobbi

**Affiliations:** 1 Department of Biochemistry and Molecular Pharmacology, Mario Negri Institute for Pharmacological Research, Milano 20156, Italy; E-Mails: mara.canovi@marionegri.it (M.C.); jacopo.lucchetti@marionegri.it (J.L.); matteo.stravalaci@marionegri.it (M.S.); paolo.bigini@marionegri.it (P.B.); mario.salmona@marionegri.it (M.S.); 2 Department of Experimental Medicine, University of Milano Bicocca, Monza 20052, Italy; E-Mail: francesca.re1@unimib.it; 3 Department of Chemistry, Materials and Chemical Engineering, Politecnico di Milano, Milano 20131, Italy; E-Mail: davide.moscatelli@polimi.it

**Keywords:** nanoparticles, surface plasmon resonance, protein corona

## Abstract

Great interest is currently being devoted to the development of nanoparticles (NPs) for biomedical purposes, designed to improve the pharmacokinetic profile of their cargos (either imaging probes or drugs) and to enhance the specific targeting at the disease site. Recent works suggest that Surface Plasmon Resonance (SPR), widely used for the analysis of biomolecular interactions, represents a technique of choice for rapid and quantitative analyses of the interaction between NPs—functionalized with specific ligands—and their putative biological targets. Moreover, SPR can provide important details on the formation and the role of the protein “corona”, *i.e*., the protein layer which coats NPs once they come into contact with biological fluids. These novel applications of SPR sensors may be very useful to characterize, screen and develop nanodevices for biomedical purposes.

## 1. Introduction

Nanotechnologies represent one of the main research endeavors of the 21st century, with potential applications in many fields. With regard to biomedical applications [1,2], great interest is currently being devoted to the development of nanoparticles (NPs) as suitable carriers for imaging probes and therapeutic agents.

NPs are supramolecular assemblies, usually ranging from 20 to 500 nm in size and generally made of phospholipids (*i.e.*, liposomes), polymers, carbon or metals, which might be designed to improve the pharmacokinetic profile of their cargos and to enhance the specific targeting at the disease site [3]. Physicochemical properties of NPs, such as size and surface characteristics, might be modulated to confer stealthness, *i.e.*, the property of escaping opsonization and macrophage-mediated clearance, enhancing NP’s circulation time [4]. Surface properties also affect the adsorption of the proteins present in biological fluids, leading to the so-called protein “corona”, which determines the pharmacokinetic and pharmacodynamic properties of NPs [5–8]. Site-specific targeting can be conveniently achieved by functionalizing the surface of NPs with suitable ligands, such as small molecules or antibodies, capable of recognizing receptors specifically located at the site of interest [9–11]. Site-specific targeting is functional to both diagnostic and therapeutic purposes, depending if NPs carry imaging probes or drugs.

Thus, the interaction of functionalized NPs with their putative biological targets, as well as the protein-NPs interactions involved in the formation of the corona, are fundamental features underlying the NPs activity and value.

Here we illustrate that these interactions can be conveniently investigated by Surface Plasmon Resonance (SPR) biosensors. SPR is a powerful label-free method widely used to study binding between two macromolecules [12–15]. Typically, one of the two interacting partners is immobilized on a sensor chip surface, and the other is flowed through a microfluidic system in contact with the chip surface. Binding is revealed in real time as a change of mass at the surface, and the interaction can be characterized in terms of on and off rates (kinetics) and binding strength (affinity) [16]. Due to these features, SPR has the potential for being a technique of choice for a rapid and quantitative *in vitro* method to characterize, screen and develop NPs for biomedical purposes.

## 2. Experimental Section

For the present SPR studies we used the ProteOn XPR36 apparatus (BioRad, Hercules, CA, USA), which has six parallel flow channels that can be used to uniformly immobilize strips of six ligands on the sensor chip gold surface. After ligand immobilization, the ProteOn XPR36 fluidic system can automatically rotate 90° [17] so that up to six different analytes can be injected simultaneously over all the immobilized ligands.

For liposome immobilization, a GLM sensor chip (BioRad) was used. Undecylamine (Sigma-Aldrich, St. Louis, MO, USA) was amine-coupled to the surface with classical amine-coupling chemistry. Briefly, the surface was activated with N-hydroxysuccinimide/1-ethyl-3- (3-dimethyilaminopropyl)carbodiimide (NHS/EDC), and undecylamine in acetate buffer (pH 5.0) was flowed for 5 min at a flow rate of 30 μL/min. The remaining activated groups were blocked with ethanolamine, followed by a pulse with NaOH and surface regeneration with CHAPS.

Liposomes were prepared as described in [18] and were composed of a sphingomyelin-cholesterol matrix (Sm-Chol, 1:1 molar ratio) mixed or not with 20 molar % of cardiolipin (CL), dimyristoylphosphatidic acid (PA), phosphatidylcholine (PC) or monosialoganglioside GM1 (GM1) [18]. The diameters of these liposomes, estimated by Dynamic Light Scattering (DLS), were very similar, being (mean ± SD) 144 ± 10 nm, 143 ± 9 nm, 151 ± 9 nm, 178 ± 10 nm and 14 1 ± 12 nm for plain, CL, PA, PC and GM1 liposomes, respectively [18]. Liposomes (1 mM total lipids) in 10 mM TRIS, 150 mM NaCl, 1 mM EDTA (pH 7.4) were flowed in the SPR apparatus for 5 min at a flow rate of 30 μL/min. Amyloid-β (Aβ) was immobilized on the sensor chip as described previously [18,19].

For NP corona studies, recombinant human low-density lipoprotein receptor-related protein-1 (LRP-1) cluster IV Fc chimera (R&D Systems, Minneapolis, MN, USA), anti-apolipoprotein E antibody (ApoE-Ab, Abcam, Cambridge, UK) and anti-human albumin antibody (HSA-Ab, GeneTex, Irvine, CA, USA) were immobilized in parallel-flow channels of a GLC sensor chip (BioRad) using amine-coupling chemistry. After chip surface activation, protein solutions were injected for 5 min at a flow rate of 30 μL/min, and remaining activated groups were blocked with ethanolamine, pH 8.0. All ligands were immobilized at a concentration of 30 μg/mL in acetate buffer, pH 4.0 for ApoE-Ab and HSA-Ab, or pH 3.0 for LRP-1. Final immobilization levels were similar for all chambers, *i.e.*, approximately 4,000 Resonance Units (1 RU = 1 pg protein/mm^2^). A reference surface was prepared in parallel using the same immobilization procedure but without addition of protein (empty surface).

Negatively charged poly(methyl methacrylate) (PMMA) NPs, with diameters of approximately 50 nm [20], were used for the present studies. NPs were incubated for 1 h in human plasma at 37 °C at a concentration of 1 × 10^12^ NPs/mL. A parallel plasma sample was incubated in the absence of NPs and another control was prepared incubating NPs in 10 mM phosphate buffer, containing 150 mM NaCl (Phosphate buffer saline, PBS). After centrifugation at 13,000 × g for 15 min, the pellets, containing NPs and/or precipitated plasmatic proteins, were resuspended in PBS containing 0.005% Tween-80 (PBST), 1000-fold the original plasma volume. These suspensions were either directly injected into the SPR instrument or centrifuged again, and the supernatant injected. After each injection, bound analytes were allowed to completely dissociate before the next injection.

## 3. Results and Discussion

### 3.1. SPR for Studying the Interaction of Functionalized NPs with Their Putative Biological Targets

**Figure 1.**
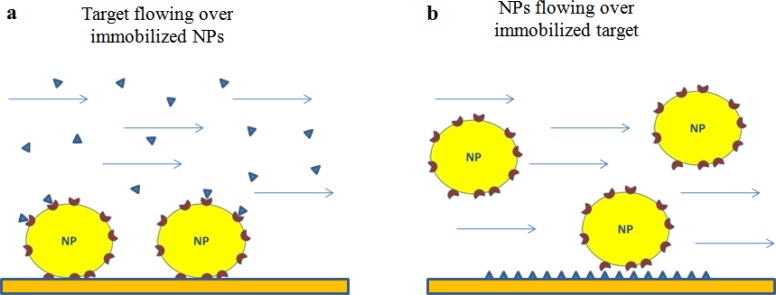
SPR approaches to study interactions between functionalized NPs and their putative biological targets. (**a**) Flowing of the target onto ligand-functionalized NPs immobilized on the sensor surface. (**b**) Flowing of ligand-functionalized NPs onto immobilized target (note the possibility that multivalent interactions underlie the binding of a single NP).

In general, two formats can be envisaged to study the interaction between ligand-functionalized NPs (Lig-NPs) and their putative targets. One involves the flowing of the target onto Lig-NPs immobilized on the sensor surface (Figure 1(a)) whereas the other involves the opposite, the immobilization of the target and the flowing of Lig-NPs (Figure 1(b)).

The literature offers examples of both formats (described below), each of them has pros and cons. In general, an higher sensitivity can be predicted with the format in Figure 1(b) due to the higher mass of Lig-NPs, but this format might be limited by the possibility to immobilize the biological target without altering its binding properties. The analysis of the data obtained with the format in Figure 1(a) are suitable to estimate unbiased kinetic constants for the interaction between the flowing target and each of the ligand molecules exposed on the NP surface; on the contrary, the binding constants estimated with the format in Figure 1(b) are likely the results of multivalent interactions occurring between each flowing Lig-NP and different immobilized target molecules (Figure 1(b)) [9,21] (see below for discussion of this important point).

#### 3.1.1. Immobilization of Functionalized NPs on the SPR Chip Surface

**Figure 2.**
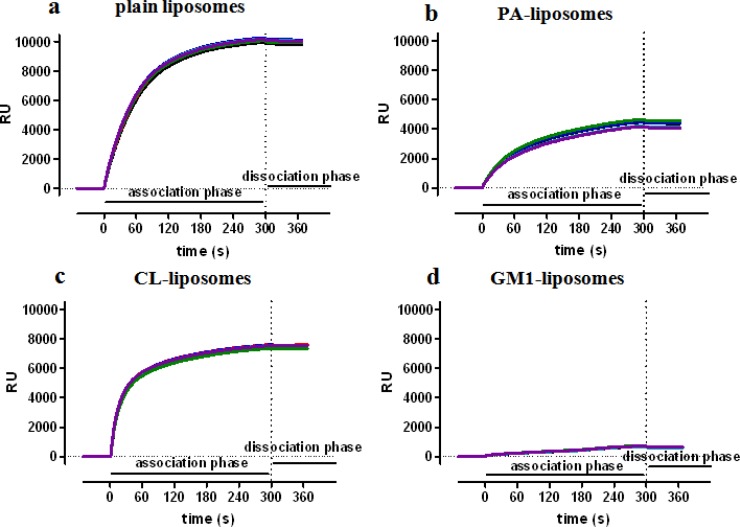
Capture of nanoliposomes on SPR chip surface. Sphingomyelin:cholesterol liposomes, including or not 20% of dimyristoylphosphatidic acid (PA, **b**), cardiolipin (CL, **c**), or monosialoganglioside GM1 (GM1, **d**) were flowed for 5 min (association phase) over a chip surface protruding lipophilic undecyl chain anchors. Panels show the raw sensorgrams, *i.e.*, the SPR signal in Resonance Units, RU, *versus* time, each normalized to a baseline value of 0. The results obtained in six parallel surfaces are shown in each panel. No decrease of SPR signal was observed during the dissociation phase, indicating a stable capture of liposomes.

NPs can be immobilized onto sensor surfaces by different approaches. Liposomes can be stably captured by sensor surfaces exposing protruding lipophilic alkyl chain anchors, which insert into the lipidic layer of the NP [22–26]. Figure 2 reports the data obtained in our lab using this approach and showing the efficient and long-lasting capture of different types of liposomes.

The maximal capture varied from 700 RU (GM1 liposomes) to 10,000 RU (plain liposomes). Since the size of the tested liposomes were very similar (≈140 nm), the differences are likely due to the lipid composition, in agreement with previous data: in particular, it had already been shown that negatively charged liposomes deposited less densely due to electrostatic repulsion [23,24]. The dissociation rate was very slow, always lower than 2 × 10^−4^ s^−1^, corresponding to less than 10% dissociation in ten minutes, and allowing the subsequent injection (binding) of analytes onto captured nanoliposomes.

Some authors [22,25], but not others [24], showed that liposomes immobilize intact onto the sensor chip. With this approach, Cooper *et al.*[22] demonstrated that captured liposomes exposing (functionalized with) the ganglioside GM1 bind flowing cholera toxin with the expected affinity.

Other procedures have been used to capture liposomes on the sensor chip [23], such as: (i) liposomes containing amounts of biotinylated lipids can be covalently attached to chip surfaces exposing avidin [27]; (ii) liposomes functionalized with antigens can be captured by the corresponding antibodies covalently attached to the sensor surface [28]; antisense DNA-derived liposomes can be retained by hybridization to sense DNA tethers attached to the chip surface [29]. In a similar manner, thiol-conjugated polymeric NPs can be covalently immobilized onto bare gold surfaces for further interaction analysis [30,31].

#### 3.1.2. Flowing of Functionalized NPs onto Biological Targets Immobilized on the Sensor Chip

Some examples of this format have been obtained in our laboratory, looking at the Aβ-binding properties of NPs functionalized with putative Aβ ligands, investigated as vectors for the targeted delivery of new diagnostic and therapeutic molecules for Alzheimer’s disease (AD). For these SPR studies, we immobilized Aβ on the chip surfaces, either as monomers or as fibrillar aggregates [19], and injected NPs in the microfluidic channels.

**Figure 3.**
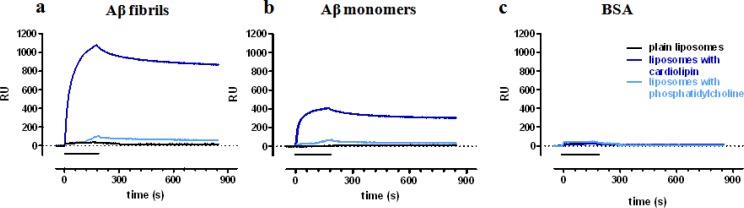
Flowing of functionalized NPs onto biological targets immobilized on SPR chip surface. Amyloid-β (Aβ) species and BSA (reference protein) were immobilized in parallel-flow channels of a sensor chip. Plain liposomes (sphingomyelin:cholesterol 1:1) or liposomes carrying 20% of cardiolipin (CL) or phosphatidylcholine (PC) were flowed for 3 min (bar), followed by 11 min dissociation phase. Panels show the raw sensorgrams, *i.e.*, the SPR signal in Resonance Units, RU, *versus* time. The presence of cardiolipin (CL-liposomes, blue lines) confers the ability to bind to immobilized Aβ, in particular Aβ fibrils (**a**), whereas no binding was observed on BSA (**c**). No or negligible binding was detected with plain liposomes (black lines) or with liposomes bearing phosphatidylcholine (PC-liposomes, light blue lines). Data from [18] with modifications.

In the first study [18] we showed that functionalization of liposomes or solid lipid nanoparticles with anionic phospholipids (such as CL) confers the ability to bind to immobilized Aβ fibrils (Figure 3(a)). Much lower, or negligible, binding was detected with plain NPs (Figure 3(a)) or with NPs exposing other phospholipids such as PC (Figure 3(a)). Furthermore, CL-NPs preferentially bound to Aβ fibrils whereas lower binding was observed on Aβ monomers (Figure 3(b)). No binding was found on bovine serum albumin (BSA, always tested in parallel as reference protein, Figure 3(c)). Analysis of these sensorgrams allowed to estimate the affinity of cardiolipin-exposing liposomes for immobilized Aβ fibrils (K_D_ of 44 nM calculated on the cardiolipin concentration) [18].

The same approach was used to investigate the Aβ-binding properties of liposomes functionalized with the putative Aβ-ligand curcumin and with a new curcumin derivative. We could observe [32] that the liposomes exposing the curcumin derivative (maintaining the planarity) bind with high affinity (K_D_ values in the low nM range) and specificity to Aβ fibrils whereas those exposing non-planar curcumin did not bind to Aβ, providing fundamental data for further developments.

Finally, SPR was used to characterize the binding properties of liposomes decorated with a new anti-Aβ monoclonal antibody (Aβ-MAb) [33]. The data showed that Aβ-MAb-liposomes, but not IgG-liposomes, markedly bound to both Aβ monomers and fibrils, but not to BSA. K_D_ values (calculated on Aβ-MAb content) were about 0.5 and 2 nM with liposomes at high and low Aβ-MAb density respectively. Aβ-MAb liposome binding to Aβ fibrils was additionally confirmed by an ultra-centrifugation technique, in which interactions occur in solution under physiological conditions. Further studies showed that the same Aβ-MAb-liposomes bound amyloid deposits in post-mortem AD brain samples, confirming the potential of these NPs for the diagnosis and therapy of AD.

In all these studies we observed that the binding of functionalized NPs to Aβ showed very low dissociation rate constant (K_off_), approaching those of a pseudo-irreversible binding (see Figure 3(a)). This observation is likely explained by the presence of multivalent interaction, *i.e*., different molecules of Aβ-ligands exposed on the same NP contribute to the binding to the immobilized Aβ (Figure 1(b)). Thus, it has been previously shown that a multivalent ligand (dendrimer [9]; NP [21]) has a binding affinity for its target which can greatly exceed, even by 3–5 orders of magnitude, the binding affinity of the same ligand, if monovalent. In agreement, we observed that the affinity for Aβ fibrils of the curcumin derivative when exposed on liposomes (nanomolar range) was much higher than the affinity of the corresponding compound not attached to liposomes (micromolar range) [32]. The binding avidity of functionalized NPs is an added value of Lig-NPs, with relevant biological implications [3,9,34], and the ability to unravel it by SPR is noteworthy.

### 3.2. SPR for Investigating Protein Corona

When NPs enter biological fluids, e.g., the blood after systemic administration or the cytosol after cell uptake, they are coated by biomolecules, in particular proteins, which adsorb onto their surface [7,8]. The new layer, or corona, confers to NPs properties which might be very different from those of the bare NPs, markedly affecting their behaviour with regards to pharmacokinetics (biodistribution, interaction with immune system and clearance) and pharmacodynamics (interaction with cells and putative biological targets) [6,7].

The formation and composition of protein corona is a complex process depending on the physicochemical properties of NPs (e.g., size, composition and charge) [5,30,35–37] and on the biological fluid (protein’s abundance and affinity for NPs surface) [7,35,38]. The protein adsorption is also a dynamic and competitive process, where more abundant proteins might adsorb at first, even with weak bonds, being displaced thereafter by less abundant proteins with higher affinity for the NPs surface [30,39,40]. For example, experimental data [41] and the results of a simulation model [40] suggest that human serum albumin (HSA) forms the early corona, and is soon replaced by higher-affinity and slower-exchanging apolipoproteins. The labile corona formed at initial time points is usually indicated as the “soft corona” and is characterized by a fast exchange rate with free proteins all around, whereas the latter is indicated as “hard corona” and is due to a more stable layer of proteins. Proteins adsorbed after incubation of NPs in plasma can be identified by proteomic analysis, and previous studies [5,8,36] indicated that the hard corona is mainly composed by 10–50 proteins including HSA, specific lipoproteins and proteins involved in coagulation and the complement pathway, with different consequences relevant to nanomedicine and nanosafety. Thus, adsorption of opsonins (IgG and complement factors) promotes phagocytosis with removal of NPs from the bloodstream [4,42,43], whereas adsorption of dysopsonins like HSA and apolipoproteins likely enhances circulation time [44,45]. Adsorbed apolipoproteins might also favor transport across the blood-brain-barrier (BBB) [46–48], likely through the interaction with low-density lipoprotein (LDL) receptors [49].

The importance of the protein corona for NPs behaviour must be carefully considered when developing and assaying new NPs, taking into account that the corona composition is dynamic and affected by the physicochemical properties of NPs. Proteomic assay, although very informative, cannot be considered a technique of choice for a rapid screen of different NPs, or for a detailed time-course study during their incubation in biological fluids. As a complementary technique we therefore envisage to apply SPR, developing new experimental formats suitable to investigate the binding of relevant proteins onto NPs, in a short time and with a relatively high-throughput.

Our strategy is to incubate NPs in the biological fluid of interest (e.g., plasma), isolate NPs by centrifugation, resuspending them in buffer. This suspension is then flowed, in the SPR channels, onto surfaces immobilizing suitable ligands, such as antibodies or other receptors. For the first, proof-of-principle studies, we selected anti-HSA and anti-ApoE antibodies (HSA-Ab and ApoE-Ab, respectively), and LRP-1 (low-density lipoprotein receptor-related protein), to investigate the NPs adsorption of HSA, ApoE and other LRP-1 binding proteins, whose relevance has been described above. For these studies we used 50 nm negatively-charged polymeric NPs.

**Figure 4.**
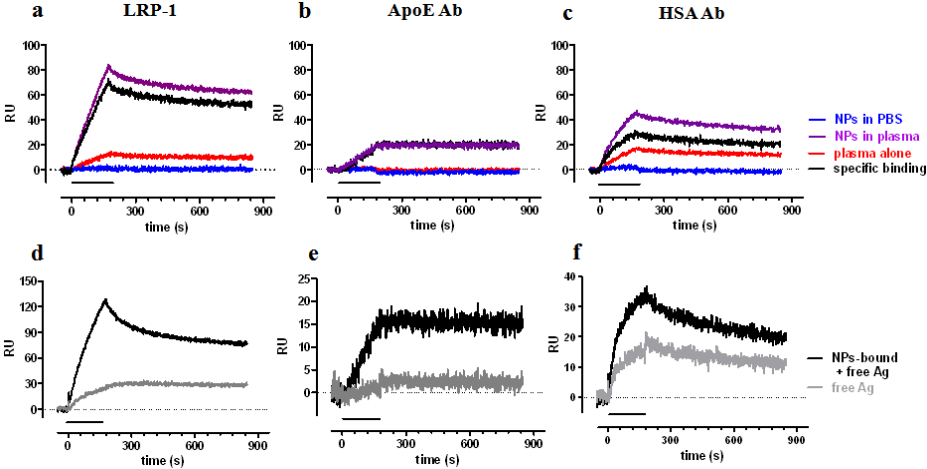
SPR study of protein corona. (**a**–**c**): PMMA NPs were preincubated in human plasma for 1 hour, precipitated by centrifugation, resuspended in buffer and injected for 3 min onto chip surfaces immobilizing LRP-1 (a), anti-ApoE Ab (b) or anti-HSA Ab (c). Purple lines show the corresponding sensorgrams. As internal controls we injected NPs samples preincubated in buffer (blue sensorgrams) or sample obtained, with the same procedure (centrifugation and resuspension) from plasma alone (red sensorgrams). Black sensorgrams were obtained as difference between the purple and the red sensorgrams, to highlight the signal specifically due to proteins adsorbed to NPs during the incubation in plasma. (**d**–**f**): Black sensorgrams were obtained as described above. Resuspended NPs samples underwent further centrifugation and supernatants were injected (gray sensorgrams). See text for details.

A clear SPR signal was detected injecting NPs which had been preincubated with human plasma for one hour, at 37 °C (Figure 4 upper panel, purple sensorgrams), whereas no signal was found with NPs preincubated in buffer alone (blue sensorgrams, indicating no binding of plain NPs to the chip surface). Some signal was found for samples in which plasma was incubated in the absence of NPs, indicating the precipitation of relevant proteins during the centrifugation step (red sensorgrams). This latter signal was then subtracted from that found in the presence of NPs, thus allowing us to estimate the SPR signal specifically due to proteins adsorbed to NPs during the incubation in plasma (black sensorgrams). The highest signal was found on the surface immobilizing LRP-1, followed by HSA-Ab and ApoE-Ab. In all cases the specific binding was quite stable with low dissociation rate constants: 3.3 × 10^−4^ s^−1^, 4.6 × 10^−4^ s^−1^ and >10^−5^ s^−1^, for LRP-1, ApoE-Ab and HSA-Ab, respectively.

The centrifugation step is necessary to remove plasmatic unbound antigens, isolating NPs-antigen complexes; however, the subsequent resuspension of the pellet might induce the dissociation of antigen from NPs and consequently the final signal (black sensorgrams) might be due to either the antigen still bound to NPs or to antigen dissociated from NPs. To evaluate this point, and to understand the stability of the antigen-NPs interaction, we centrifuged samples again, to pellet NPs, and injected the supernatants (containing dissociated antigen only). The corresponding sensorgrams are shown, as gray lines, in the lower panels of Figure 4, in comparison with the sensorgrams obtained before the second centrifugation (black lines). These data indicate that dissociated antigen account for a small proportion of the binding to ApoE-Ab and LRP-1, suggesting that these antigens firmly bind to NPs. On the contrary, half of the binding to HSA-Ab is due to dissociated HSA, indicating a weaker binding between HSA and NPs, in agreement with previous observations [41] and simulation analysis [40].

## 4. Conclusions

In summary, our studies and others in the literature confirm that SPR may be the technique of choice for the rapid and informative evaluation of relevant properties of NPs, useful for screening and development purposes. Thus, SPR may provide a convenient *in vitro* approach to confirm the ability of functionalized NPs to bind to their putative biological target, and different approaches can be used to achieve this objective. A rapid *in vitro* assay is useful since the attachment of the functionalizing ligand to NPs might impair its binding properties [32] and these studies can also unveil an increased affinity for the target due to multivalent interactions [9,18,21]. Moreover, the SPR approach allows a rapid evaluation of the binding specificity.

We also provide evidence that SPR is a fast method to investigate the adsorption of selected proteins onto NPs surface (corona). In the present study we looked at the adsorption of HSA because of possible consequences on NPs circulation time, and LRP-1-binding proteins and ApoE because of their involvement for BBB passage [46–51]. Thus, the SPR assay could serve, for example, to rapidly predict, *in vitro*, the potential brain uptake of different NPs. The analysis for other antigens might allow us to predict other NPs behaviour, such as cell uptake or interaction with the immune system. Importantly, SPR also allows a relatively high throughput analysis, permitting systematic screenings of the behaviour of different NPs (e.g., different size, charge, composition, *etc.*), after incubation for different periods of time in different biological fluids. This is further added value of SPR, potentially very useful in the process of NPs development.
